# Support needs, received support and mental well-being in Austrian farmers: a mixed-methods approach

**DOI:** 10.3389/fpubh.2025.1600624

**Published:** 2025-06-30

**Authors:** Elke Humer, Afsaneh Gächter, Christoph Pieh, Viktoria Neubauer

**Affiliations:** ^1^Department for Psychosomatic Medicine and Psychotherapy, University of Continuing Education (Danube University Krems), Krems an der Donau, Austria; ^2^Faculty of Psychotherapy Science, Sigmund Freud University Vienna, Vienna, Austria; ^3^FFoQSI GmbH – Austrian Competence Centre for Feed and Food Quality, Safety and Innovation, Tulln, Austria; ^4^Centre for Food Science and Veterinary Public Health, Clinical Department for Farm Animals and Food System Science, University of Veterinary Medicine Vienna, Vienna, Austria

**Keywords:** mental health, support wishes, Austrian agriculture, received support, well-being

## Abstract

**Introduction:**

Although Austrian farmers are at increased risk for mental ill health, there is limited research on their specific support needs and hardly any evidence on the prevalence of these support requirements. This study aims to explore the mental health support needs of Austrian farmers and to identify the types of support they consider most useful.

**Methods:**

An online survey was conducted among 2,006 Austrian farmers. Participants completed standardized questionnaires assessing mental health parameters—including the PHQ-9 (depression), GAD-7 (anxiety), ISI-2 (insomnia), PSS-4 (perceived stress), WHO-5 (well-being), and CAGE (alcohol abuse)—and answered a dichotomous question regarding their desire for mental health support. Those who indicated a wish for support were invited to provid free-text descriptions of the specific type of help they desired. Additionally, farmers were asked whether they were already receiving support to improve their mental well-being. A mixed-methods approach was used to analyze both the quantitative mental health data and the qualitative free-text responses.

**Results:**

Approximately 32% of the farmers expressed a desire to receive support to improve their mental well-being. Qualitative analysis of the free-text responses revealed a variety of support wishes, with coaching, counselling, mediation, and psychotherapy being the most common. Other prominent themes were the need for practical support related to farm work, time for recreation, financial support, and enhanced communication. Furthermore, around 14% of participants reported already receiving some form of mental health support. Farmers who either desired or were receiving support exhibited higher levels of depression, anxiety, insomnia, alcohol abuse, and perceived stress compared to those without such support needs.

**Conclusion:**

The study identifies a vulnerable subgroup within the Austrian farming community that experiences significant mental health challenges and expresses clear support needs. These findings emphasize the importance of developing tailored interventions that address both the psychological and practical aspects of farmers’ well-being, thereby enhancing resilience and improving overall mental health outcomes in this essential occupational group.

## 1 Introduction

The agricultural sector remains a cornerstone of many economies worldwide, yet the mental health of farmers has long been an underrecognized public health concern (1). Farmers are routinely exposed to a unique constellation of stressors—including financial uncertainty, unpredictable weather conditions, and increasing bureaucratic demands—that contribute to elevated levels of psychological distress (2–4). These challenges are further compounded by the ongoing transformation of the agricultural sector, marked by globalization, climate change, and structural changes in rural economies (5). Despite growing awareness of these issues in international research, the specific support wishes of farmers and their help-seeking behavior remain insufficiently explored.

Farmers often work in isolated environments with limited access to social support and mental health services, which can exacerbate feelings of loneliness and stress (6). Numerous studies have reported that agricultural workers exhibit higher rates of depression, anxiety, alcohol abuse, and stress compared to the general population (1, 7, 8). These mental health challenges are frequently compounded by economic pressures, such as fluctuating market prices and the high costs of agricultural inputs, which not only affect the viability of farming operations but also the personal well-being of the farmers (9). In many cases, these factors interact to create a situation where farmers feel both overwhelmed and isolated, with few avenues for effective support. Most studies on the mental health of farmers have been conducted in the United States (1), where farming conditions and stressors may differ substantially from those in smaller-scale, sustainability-oriented systems like Austria’s.

In Austria, the agricultural sector is shaped by a high degree of regional and structural diversity. Farming practices range from small, family-owned farms to more industrialized operations, with a particularly strong prevalence of small-scale farming and the highest share of organic agriculture within the European Union (10). The country’s mountainous topography and decentralized settlement structure pose unique challenges to agricultural work, infrastructure, and service accessibility. Austria is internationally recognized for its high standards in animal welfare and environmental protection in agriculture. Compared to the United States, for instance, Austria has significantly stricter regulations. For example, the use of cages for laying hens has been banned in Austria since 2009, whereas cage systems are still common in the U. S. (11). In terms of environmental protection, Austria has one of the highest shares of organic farmland in the world—over 25%—supported by national agri-environmental programs such as ÖPUL (12). Due to the high number of small farms, capital needs are high, and economies of scale limited. Many farmers rely on public subsidies, which are becoming scarcer. To remain viable, small farms—especially in less favorable regions—must diversify through direct marketing, tourism, and non-agricultural activities (13). These contextual differences underline the importance of region-specific investigations, as findings from international studies may not fully capture the lived realities and support needs of Austrian farmers.

Recent research has shown that approximately one-third of the general population in Austria expresses a desire for mental health support (14). However, evidence regarding the prevalence of such support wishes among farmers is scarce. Preliminary data indicate that a significant proportion of farmers experience mental health challenges, yet only a minority have accessed professional support services (4). This discrepancy raises critical questions about the barriers to help-seeking in this group. Factors such as the stigma associated with mental health issues, a perceived lack of services tailored to the agricultural context, and practical barriers—like long working hours and geographic isolation—may contribute to the underutilization of available support (15, 16).

Considering these observations, the present study seeks to advance our understanding of mental health-related support needs among Austrian farmers. Using a mixed-methods design, the study aims to capture both the prevalence and nature of support wishes, explore their associations with mental health parameters, and identify sociodemographic and contextual predictors of both support wishes and actual support utilization. The overarching goal is to inform the development of comprehensive, accessible, and culturally appropriate support strategies that are attuned to the specific needs and life realities of this vulnerable occupational group.

The study was guided by the following research questions:

1. What sociodemographic factors (e.g., gender, age, region, education, financial situation, marital status, farm employment type) are associated with the expression of a support wish among Austrian farmers?
2. How do mental health parameters—including depression, anxiety, insomnia, alcohol abuse, and stress—differ between farmers who express a wish for support and those who do not?
3. What specific types of support are most frequently desired by Austrian farmers and are there differences with respect to gender?
4. What sociodemographic factors (e.g., gender, age, region, education, financial situation, marital status, farm employment type) are associated with the utilization of support among Austrian farmers?
5. How do mental health parameters—including depression, anxiety, insomnia, alcohol abuse, and stress—differ between farmers who utilize mental health support and those who do not?

## 2 Materials and methods

### 2.1 Study design

Between October 4, 2024, and February 28, 2025, an online survey was conducted among Austrian farmers using the LimeSurvey platform (LimeSurvey GmbH, Hamburg, Germany). Participation was completely voluntary, and no monetary or material incentives were offered. Farmers were reached through multiple channels—including announcements by agricultural chambers, farmer associations, and unions, as well as advertisements in agricultural magazines, newspapers and web pages. Additionally, promotion was supported by the Austrian Ministry of Agriculture, dairy cooperatives, breeding associations, and other agricultural organizations. In total, 2,006 farmers fully completed the questionnaire, which corresponds to a completion rate of 72.3% from the 2,773 individuals who accessed the survey link.

This study was carried out in accordance with the principles of the Declaration of Helsinki and received approval from the Ethics Commission of the Faculty of Psychotherapy Science at Sigmund Freud University Vienna, Austria (ethical approval number: XCXFA65WBWE@5490500, approved on November 20, 2023). All participants provided electronic informed consent to take part in the study.

### 2.2 Measures

#### 2.2.1 Wish for support

To assess mental health support needs, participants first responded to a yes/no question asking whether they desired support to improve their mental well-being.

Those who answered “yes” were then presented with an open-ended question asking them to describe the type of support they believed would be beneficial.

#### 2.2.2 Utilization of support

Participants were also asked whether they are currently receiving help to improve their mental well-being. This was captured using a dichotomous yes/no question.

#### 2.2.3 Sociodemographic characteristics

Participants provided demographic details including gender (male, female, diverse), age (in years), education (no formal education, secondary school, apprenticeship, vocational secondary school, higher secondary school, university), region (federal states of Austria), and relationship status (single or partnered). In addition, the type of farm employment (full-time or part-time) and the subjective assessment of their financial situation (ranging from very good to very poor) were assessed.

#### 2.2.4 Mental health characteristics

The study utilized several validated instruments to assess various aspects of mental health. The WHO-5 Well-being Index (17), consisting of five items rated from 0 to 5, produces a percentage score (0–100) after multiplication by four, with scores ≤50 indicating poor well-being and potential depressive symptoms (Cronbach’s *α* = 0.85). Depressive symptoms were further measured by the PHQ-9 (18), a nine-item tool on a 0–3 scale yielding scores between 0 and 27, where scores ≥10 denote moderate depression (*α* = 0.87). Anxiety levels were assessed using the GAD-7 (19), which includes seven items scored from 0 to 3, with a cut-off of ≥10 for moderate anxiety (α = 0.91). Sleep quality and insomnia were evaluated via the ISI-2 (20), a five-point scale instrument (0–4) with scores of 6 or higher indicating moderate insomnia (*α* = 0.71). The Perceived Stress Scale (PSS-4) (21), with four items rated from 0 to 4, uses a cut-off of ≥6 to define moderate stress levels (α = 0.82). Finally, alcohol misuse was screened with the CAGE questionnaire (22), comprising four yes/no questions, where two or more affirmative responses suggest problematic alcohol consumption (*α* = 0.60).

### 2.3 Data analysis

#### 2.3.1 Quantitative analysis

Chi-square tests and *t*-test were used to characterize potential differences in the sociodemographic features of the wish for support and no wish for support group as well as the receipt of support and no receipt of support group. The mental health parameters, evaluated with the questionnaires WHO-5, PHQ-9, GAD-7, ISI, CAGE, and PSS-4, were compared between the two “support wish” group (yes/no) and the “receipt of support” groups (yes/no) using the Chi-square test.

*P*-values ≤ 0.05 were considered as statistically significant. For the statistical analyses IBM SPSS Statistics version 29.0. (IBM Corp., Armonk, New York, USA) was used.

#### 2.3.2 Qualitative data analysis and mixed method analysis

To analyze the free-text responses on support wishes, a conventional content analysis was applied (23). All valid free-text responses (*n* = 393) were subjected to an inductive content analysis approach including coding and categorization. No selection criteria were applied beyond completeness of data. Initially, the responses—ranging from single keywords to full sentences—were thoroughly reviewed to gain an overall understanding of the data. An initial coding system was developed and subsequently refined through discussions to resolve any uncertainties, leading to a set of clear categories and subcategories. Inductive codes were generated from the responses, and those that did not fit into the predefined categories were later grouped under an “others” category. This systematic process allowed for the quantification of key themes, which were then integrated into the quantitative and mixed-method analyses. To analyze potential gender-differences, the frequencies of the answers in each category and subcategory were compared between female and male farmers using Chi-square tests. As only one gender-diverse farmer provided a free-text answer, this individual was excluded from statistical analyses of gender-related differences.

## 3 Results

### 3.1 Sample and study sample characteristics

A total of 2,006 participants attended the survey and 633 participants (31.6%) answered the question “Would you like to receive support to improve your mental health?” with “yes.” Out of the sample of participants stating a wish for support, 240 participants did not elaborate on their answer to the follow-up question “Please describe which kind of support would be helpful for you.” In more detail, within these 240 participants, 234 participants provided no answer by leaving the field empty, while 6 participants entered some random letters or punctuation. Thus, out of the “support wish” group (*n* = 633) a total of 393 participants (62.1%) described their wishes in more detail (Table 1).

**Table 1:** The category system that emerged from the responses to the free-text question “Please describe which kind of support would be helpful for you” from the participants stating to wish support to improve their mental health status.

Categories	*N*	% of participants wishing support (*n* = 633)	% of participants providing an answer (*n* = 393)
Professional help except professional mental support wishes	122	19.3	31.0
Coaching/Counseling/Mediation	66	10.4	16.8
Farm help	38	6.0	9.7
Care help	6	0.9	1.5
Domestic help	5	0.8	1.3
Medical support	5	0.8	1.3
Professional mental support	84	13.1	21.4
Free	18	2.8	4.6
Easily accessible	10	1.6	2.5
Anonymous	2	0.3	0.5
Work-related wishes	79	12.5	20.1
Agrar policy/bureaucracy	45	7.1	11.5
Appreciation	15	2.4	3.8
Workload	14	2.2	3.6
Recreational activities	59	9.3	15.0
Financial support	52	8.2	13.2
Communication	50	7.9	12.7
Do not know	19	3.0	4.8
Self-care and stress management	18	2.8	4.6
Family support	13	2.1	3.3
Others/not specified	11	1.7	2.5
Already receive help	4	0.6	1.0

Table 2 provides a detailed description of the sociodemographic characteristics and difference between the “support wish” (*n* = 633) and the “no support wish” group (*n* = 1,373). The “support wish” group comprised a similar proportion of female (47.2%) and male (52.6%) farmers, whereas the proportion of male farmers was considerably higher in the no-support wish group (65.5%). Age did not differ between the two groups. Significant differences became evident for the region, where within the support-wish group the proportion of farmers from Eastern and Southern Austria was higher and that of farmers from Western Austria was lower compared to the no-support wish group. Also, differences with respect to education attainment became evident, with a lower proportion of farmers with no education/secondary school or apprenticeship, but a higher proportion of farmers with a university degree wished for support. The difference between those two groups was also evident in terms of financial situation. In the “support wish” group a higher proportion of farmers reported a poor or very poor financial situation compared to the “no support wish” group. No differences between both groups emerged for marital status and farm employment status.

**Table 2:** Study sample characteristics of the wish for support group (*N* = 633) and the no wish for support group (*N* = 1,373).

Sociodemographic Characteristics	Support wish	No support wish	Statistics
	% (N)	% (N)	
Gender			
Female	47.2 (299)	34.5 (473)	χ^2^ (2) = 30.40 *p* < 0.001
Male	52.6 (333)	65.5 (899)	
Diverse	0.2 (1)	0.1 (1)
Age			
18–24	1.4 (9)	1.3 (18)	χ^2^ (5) = 8.432 *p* = 0.134
25–34	17.4 (110)	15.7 (215)	
35–44	29.4 (186)	31.6 (434)	
45–54	33.8 (214)	29.3 (402)	
55–64	16.7 (106)	20.1 (276)	
≥65	1.3 (8)	2.0 (28)	
Age in years (average, ± SD)	45.25 (10.39)	44.59 (10.11)	t (2004) = 1.346; *p* = 0.178
Region			
Western Austria	69.8 (442)	75.7 (1,039)	χ^2^ (2) = 8.016 *p* = 0.018
Eastern Austria	19.6 (124)	16.4 (225)	
Southern Austria	10.6 (67)	7.9 (109)	
Education attainment level			
No education/secondary school	1.6 (10)	2.5 (35)	χ^2^ (4) = 13.084 *p* = 0.011
Apprenticeship	16.9 (107)	20.7 (284)	
Vocational secondary school	45.7 (289)	45.2 (621)	
Higher secondary school	21.8 (138)	22.0 (302)	
University	14.1 (89)	9.5 (131)	
Financial situation			
Very good	6.5 (41)	8.5 (117)	χ^2^ (4) = 27.47 *p* < 0.001
Good	29.1 (184)	33.5 (460)	
Moderate	35.2 (223)	37.4 (513)	
Poor	19.3 (122)	16.0 (219)	
Very poor	10.0 (63)	4.7 (64)	
Marital status			
Single	11.2 (71)	9.7 (133)	χ^2^ (1) = 1.110 *p* = 0.292
In a relationship	88.8 (562)	90.3 (1,240)	
Farm employment status			
Full-time farming	68.9 (436)	64.5 (885)	χ^2^ (1) = 3.7660 *p* = 0.052
Part-time farming	31.1 (197)	35.5 (488)	

The regions were classified according to NUTS 1 (Nomenclature of territorial units for statistics) into three major socio-economic regions (Eastern Austria: Burgenland, Lower Austria, Vienna; Southern Austria: Carinthia, Styria; Western Austria: Upper Austria, Salzburg, Tyrol, Vorarlberg). The *p*-value is given as a two-tailed value.

A total of 281 participants (14.0%) answered the question “Do you already received support to improve your mental health?” with “yes.” Table 3 provides a detailed description of the sociodemographic characteristics and difference between the “support receiver” (*n* = 281) and the “no support receiver” group (*n* = 1,725). The “support receiver” group comprised a similar proportion of female (52.3%) and male (47.7%) farmers, whereas the proportion of male farmers was considerably higher in the no-support receiver group (63.7%). The age and region did not differ within both groups. Differences with respect to education attainment became evident, with a lower proportion of farmers with no education/secondary school, apprenticeship, or vocational secondary school, but a higher proportion of farmers with higher school or university attainment already received support. No differences between both groups emerged for financial situation, marital status, and farm employment status.

**Table 3:** Study sample characteristics of the receiving support group (*N* = 281) and the no support group (*N* = 1,725).

Sociodemographic Characteristics	Support	No support	Statistics
	% (N)	% (N)	
Gender			
Female	52.3 (147)	36.2 (625)	χ^2^ (2) = 26.59 *p* < 0.001
Male	47.7 (134)	63.7 (1,098)	
Diverse	0.0 (0)	0.1 (2)
Age			
18–24	1.1 (3)	1.4 (24)	χ^2^ (5) = 3.322 *p* = 0.651
25–34	18.9 (53)	15.8 (272)	
35–44	31.7 (89)	30.8 (531)	
45–54	30.2 (85)	30.8 (531)	
55–64	17.1 (48)	19.4 (334)	
≥65	1.1 (3)	1.9 (33)	
Age in years (average, ± SD)	44.32 (10.09)	45.16 (10.34)	t (2004) = −1.263; *p* = 0.207
Region			
Western Austria	70.1 (197)	74.4 (1,284)	χ^2^ (2) = 2.267 *p* = 0.322
Eastern Austria	20.6 (58)	16.9 (291)	
Southern Austria	9.3 (26)	8.7 (150)	
Education attainment level			
No education/secondary school	0.4 (1)	2.6 (44)	χ^2^ (4) = 20.70 *p* = 0.011
Apprenticeship	16.7 (47)	19.9 (344)	
Vocational secondary school	40.2 (113)	46.2 (797)	
Higher secondary school	26.0 (73)	21.3 (367)	
University	16.7 (47)	10.0 (173)	
Financial situation			
Very good	7.1 (20)	8.0 (138)	χ^2^ (4) = 4.258 *p* = 0.372
Good	32.0 (90)	32.1 (554)	
Moderate	33.1 (93)	37.3 (643)	
Poor	20.6 (58)	16.4 (283)	
Very poor	7.1 (20)	6.2 (107)	
Marital status			
Single	10.7 (30)	10.1 (174)	χ^2^ (1) = 0.092 *p* = 0.292
In a relationship	89.3 (251)	89.9 (1,551)	
Farm employment status			
Full-time farming	67.6 (190)	65.6 (1,131)	χ^2^ (1) = 0.452 *p* = 0.501
Part-time farming	32.4 (91)	34.4 (594)	

The regions were classified according to NUTS 1 (Nomenclature of territorial units for statistics) into three major socio-economic regions (Eastern Austria: Burgenland, Lower Austria, Vienna; Southern Austria: Carinthia, Styria; Western Austria: Upper Austria, Salzburg, Tyrol, Vorarlberg). The *p*-value is given as a two-tailed value.

### 3.2 Mental health parameters and their wish for support

Participants with support wishes had an increased prevalence of scores in depression, anxiety, insomnia, and high stress above clinical cut-offs. Throughout all tested parameters, participants wishing for support significantly exceeded the cut-off values more frequently compared to those claiming no support wish, which became most evident for depressive symptoms (46.3% vs. 19.5%) and anxiety symptoms (49.8% vs. 22.5%) (Table 4).

**Table 4:** Comparison of participants above the cut-off values for the mental health parameters and their wish for support.

Above cut-off^a^	Support wish (*N* = 633)		No support wish (*N* = 1,373)		Statistics
	N	%	N	%	
Depression (PHQ-9)	293	46.3	268	19.5	χ^2^ = 154.1 *p* < 0.001
Anxiety (GAD-7)	315	49.8	309	22.5	χ^2^ = 150.2 *p* < 0.001
Insomnia (ISI-2)	144	22.7	162	11.8	χ^2^ = 40.18 *p* < 0.001
Alcohol Abuse (CAGE)	133	21.0	183	13.3	χ^2^ = 19.27 *p* < 0.001
Stress (PSS-4)	528	83.4	815	59.4	χ^2^ = 113.3 *p* < 0.001
Well-being (WHO-5)^a^	435	68.7	644	46.9	χ^2^ = 82.96 *p* < 0.001

The *p*-value is given as a two-tailed value. Cut-off values were determined as follows: PHQ-9 and GAD-7 ≥ 10, ISI ≥ 6, PSS-4 ≥ 6, CAGE with more than 2 answers with “yes,” WHO-5 ≤ 50 (0–50 indicates poor well-being).

a Except for WHO-5 where scores below cut off indicate a poor well-being.

### 3.3 Mental health parameters and the receipt of support

Throughout all tested parameters, participants receiving support significantly exceeded the cut-off values for clinically relevant mental health symptoms more frequently compared to those claiming no support wish, which became most evident for depressive symptoms (39.5% vs. 26.1%) and anxiety symptoms (43.1% vs. 29.2%) (Table 5).

**Table 5:** Comparison of participants above the cut-off values for the mental health parameters and their receipt of support.

Above cut-off^a^	Support (*N* = 281)		No support (*N* = 1,725)		Statistics
	N	%	N	%	
Depression (PHQ-9)	111	39.5	451	26.1	χ^2^ = 21.59 *p* < 0.001
Anxiety (GAD-7)	121	43.1	503	29.2	χ^2^ = 21.79 *p* < 0.001
Insomnia (ISI-2)	56	19.9	250	14.5	χ^2^ = 5.524 *p* = 0.019
Alcohol Abuse (CAGE)	58	20.6	258	15.0	χ^2^ = 5.883 *p* = 0.015
Stress (PSS-4)	211	75.1	1,132	65.6	χ^2^ = 9.785 *p* = 0.002
Well-being (WHO-5)^a^	176	62.6	903	52.3	χ^2^ = 10.285 *p* = 0.001

The *p*-value is given as a two-tailed value. Cut-off values were determined as follows: PHQ-9 and GAD-7 ≥ 10, ISI ≥ 6, PSS-4 ≥ 6, CAGE with more than 2 answers with “yes,” WHO-5 ≤ 50 (0–50 indicates for poor well-being).

a Except for WHO-5 where scores below cut off indicate a poor well-being.

### 3.4 Specified support wishes

A total of 393 participants out of the “support wish” group (*n* = 633) specified their wish for support in the open-ended question.

Gender-differences emerged (χ^2^ = 4.944; *p* = 0.026), with a higher proportion of women (66.6%) compared to men (58.0%) within the “support wish” group, providing an open text answer to the question about specific support wishes.

Eighty three participants expressed more than one wish for support in their answer, thus the proportion of all responses subsumed in the category system exceeds a total percentage of 100%. The qualitative data analysis resulted in 11 main categories and 11 sub-categories (Table 1). Gender-differences are only reported for the specific categories, where statistical significance was reached.

#### 3.4.1 Professional help except professional mental support wishes

The largest category for improving mental well-being encompassed different types of professional support (professional mental support wishes excluded), which were identified by *n* = 122 (19.3%) participants stating to wish support.

This category was mentioned significantly more frequently by women (37.2% of female farmers providing an answer to the free text question) vs. men (24.9% of male farmers providing an answer to the free text question; χ^2^ = 6.932; *p* = 0.008).

This category was composed of the following subcategories: The largest sub-category with *n* = 66 (10.4%) was the desire for coaching, mediation, or counseling. Several participants wished for counseling for specific farm-related areas, as expressed by Respondent (R) 437, “*Every 1–3 years a kind of “coaching”—how is the farm and its people doing, what needs to be considered, what should not be overlooked, where do you want to go next year—holistic view of the farm + manager*.” The second subcategory was farm help, which was named by n = 38 (6.0%) participants. Farmers were worried about the high burden of agricultural work and wished for helping hands, such as R1518: “*It would be good if there were more people working as farm helpers to run or support the farm during vacation or illness. I think that would also be good for the psyche of the farmers, just to know that someone is there if they get injured, for example. Even if farmers want to go on vacation, someone should be there, and the farm help should also be affordable.*” The third subcategory related to the care of other people living on the farm, such as parents, siblings, or children, which was mentioned by *n* = 6 (0.9%) farmers, such as R2025 noted “*Support in caring for my parents*.” The fourth subcategory was domestic help, which was mentioned by *n* = 5 (0.8%) participants. This subcategory was mentioned significantly more frequently by women (2.5% of female farmers providing an answer to the free text question) vs. men (0% of male farmers providing an answer to the free text question; χ^2^ = 4.912; *p* = 0.027). Furthermore, *n* = 5 (0.8%) reported that they would like medical support, such as R2266 noted: “*Faster medical help to get well again instead of waiting weeks for appointments*.”

#### 3.4.2 Professional mental support

The second-largest common need to support farmers’ well-being was professional mental support, mentioned by *n* = 84 (13.1%) respondents. Most statements were rather short such as “psychotherapy.” Three subcategories emerged. The largest subcategory, with *n* = 18 (2.8%), was related to the financial aspect of professional mental healthcare. Respondents expressed a wish for affordable or free psychotherapy, such as R 1078: “*Just as doctors are there for the body on a health insurance basis, this would also be important for psychologists; private doctors are extremely expensive.*” Another subcategory related to the wish for easier access to psychotherapy, like lower bureaucratic demands, which was desired by *n* = 10 (1.6%) participants. As an example, R1526 stated “Uncomplicated assistance, be it medical (quick mental health care without having to fill out 100 pages of applications).” A third subcategory, named by *n* = 2 (0.3%) related to the anonymity of professional mental health care.

#### 3.4.3 Work-related wishes

The third largest main category concerned work-related wishes (*n* = 79, 12.5%) and comprised three subcategories. The first subcategory related to burden by regulations, worries related to agricultural policy and bureaucratic burden. A total of *n* = 45 participants expressed wishes related to this category (7.1%), such as R 2708: “*Clear framework conditions, at least a policy that can somewhat guarantee that we are not on the verge of a price collapse for agricultural products. Despite all my love for my job, it’s only enjoyable if you achieve financial independence through all the work and make further improvements to the farm. At the moment, you just hope that no agricultural machinery suffers major damage, you would have to finance the repair or new purchase through a bank, which could quickly lead to financial instability*.” The second subcategory, reported by *n* = 15 (2.4%) farmers referred to a wish for more appreciation of their work as R 205 expressed, “*Farmers need to be valued more in society again; animal welfare is currently more important than human welfare! If the farmer is doing well, then the animals are doing well too.*” The third subcategory referred to the high workload of farmers, which was mentioned by n = 14 (2.2%), such as R2256:” *Not having to work from 1.1- to 31.12- as in the last 15 years. Never having a day off.*”

#### 3.4.4 Recreational activities

This main category, recreational activities, reported by *n* = 59 (9.3%), refers to taking a holiday to relax, creating leisure time, or just having “*short breaks from everyday live*” (R 1227).

This category was mentioned significantly more frequently by women (19.1% of female farmers providing an answer to the free text question) vs. men (10.9% of male farmers providing an answer to the free text question; χ^2^ = 5.171; *p* = 0.023).

Several farmers stated that they wished for time for health days or health restore stays. A typical answer falling within this category was provided by R 2214: “*Time off whether it’s a vacation with my husband or a health week… but that’s not possible in terms of time and there are no helpers…*”

#### 3.4.5 Financial support

A total of *n* = 52 (8.2%) of participants mentioned financial support as being useful to improve their mental health status. Some statements expressed a wish for more appreciation of their work through “*Fairer pay*” (R 1351), such as R 1528 “*Fair market conditions and a real chance to earn an income again. Everything is broken (tractor, roof at the hall, etc.)*.” Several farmers mentioned that with higher income it would be possible to find time for recreation, such as R2230 noticed “*Vacation pay to be able to pay someone to look after the farm when you want to go on vacation. The burden of always having to be there 365 days a year is very great.*”

#### 3.4.6 Communication

The next main category, reported by *n* = 50 (7.9%), was communication. The participants wished to have some kind of exchange, to be able to communicate or to confide their problems or concerns to someone. For most participants, it seemed to be important to have someone to talk to, as described by R 65 “*Conversations to get everything off your chest*.” Some participants stated that they wish to talk to friends, such as R 269, “*Does not necessarily have to be professional help, real friends, profound and trusting conversation partners, recognition would already be worth a lot.*” or expressed a wish for more social contacts, such as R 331 “*A real friend to talk to and share activities*.”

#### 3.4.7 Do not know

A significant proportion of participants (3.0%, *n* = 19) expressed that they have no idea what could help them to improve their well-being. Most statements were very brief, such as “Do not know,” whereas others expressed some degree of despair, such as “*I do not really know that either. I just sometimes have the feeling of being overwhelmed by everything*.” (R 2724) or “*Do not see any possibility*” (R 862).

#### 3.4.8 Self-care and stress management

Further *n* = 18 (2.8%) mentioned statements related to “self-care.” These participants expressed a wish for more time for own needs, methods to reduce stress and to be able to switch off and to find a more positive attitude. As an example, R 1775 stated “*Stress management in agriculture, the ability to put aside unimportant things in order to concentrate on the important things in everyday life!*”.

#### 3.4.9 Family support

A further main category that could contribute to the improvement of mental well-being was the wish for family support, which was described by *n* = 13 (2.1%) respondents. Examples are R1419 “*Family support*,” R 1390 “*Children with interest*” or R 2655 “*If a family member could work on the farm at home*.”

#### 3.4.10 Others/not specified

The main category Others/not specified, reported by *N* = 11 (1.7%), was selected when the respondents gave no specific input regarding what could be done to improve mental well-being (R 1813 expressed: “*Relief at every level*”).

#### 3.4.11 Already receive support

Finally, a total of *n* = 4 (0.6%) participants stated that they already receive support, whereby they did not specify which kind.

## 4 Discussion

The present study provides important insights into the mental health support needs of Austrian farmers, highlighting both quantitative and qualitative aspects of their support wishes and experiences. Notably, nearly one-third of all participants expressed a wish for support to improve their mental well-being. This finding reflects a considerable perceived need for psychological or psychosocial assistance in this occupational group and underscores the necessity of targeted mental health interventions tailored to the specific life and work conditions of farmers.

Consistent with previous literature indicating high mental health burden among agricultural workers (7, 8), our findings reveal a strong association between support wishes and clinically relevant levels of psychological distress. Farmers who reported a wish for support showed substantially higher proportions of depression, anxiety, insomnia, stress, alcohol abuse, and reduced well-being than those without such a wish. These results not only confirm the mental health challenges faced by farmers but also suggest that many individuals experiencing significant psychological distress are wishing for ways to manage or alleviate their symptoms.

Individuals who already received support also demonstrated higher prevalence of psychological symptoms across all measured domains compared to those not receiving support. While at first glance this may seem paradoxical, it is important to interpret these findings within the context of help-seeking behavior. It is likely that those who experience higher levels of mental health symptoms are more motivated to seek support or are more likely to have been referred to mental health services. Thus, the observed associations may reflect a selection effect, where individuals with greater mental health needs are more likely to receive support. Nonetheless, these findings reinforce the notion that a substantial subgroup of the farming population is at risk of clinically relevant psychological burden, and that current support systems are reaching only a small part of this group.

Sociodemographic analyses provide additional insights into potential disparities in help-seeking and support access among farmers. Gender differences emerged across several dimensions related to mental health support among farmers. While men were overrepresented in the overall sample, women were proportionally more likely to express a wish for support, particularly in the form of open-ended responses, and were also more likely to receive mental health support. These findings are in line with the extensive literature documenting gender differences in mental health stigma and help-seeking (24, 25), suggesting that women are more likely than men to acknowledge mental health needs and seek support (26, 27). Emerging qualitative research further deepens this understanding by illustrating how sociocultural norms—particularly in rural settings—shape farmers’ perceptions of mental health. A recent study among Irish farmers identified core barriers to help-seeking, including ideals of resilience, the notion of the “good farmer,” and pervasive stigma, especially among older men (28). These findings resonate with our own results, underscoring how traditional masculine norms and a culture of stoic self-reliance can act as powerful deterrents to accessing mental health support. Within such sociocultural frameworks, help-seeking may be perceived as weakness, contradicting the identity of the competent, self-sufficient farmer. Public health efforts aimed at promoting mental well-being in agricultural populations must therefore not only improve access to care but also address the deeper cultural narratives that inhibit help-seeking—particularly among male farmers. Interventions that are context-sensitive and engage with the farming identity (e.g., reframing help-seeking as a form of resilience or responsibility) may be especially effective in reducing stigma and enhancing uptake.

Further, differences were observed in educational attainment and financial situation, while no age differences were observed. Farmers with higher educational levels were more likely to express a wish for or to have already received support. Higher education may foster greater health literacy (29), including better recognition of mental health symptoms and reduced stigma, thus facilitating help-seeking behavior. Conversely, financial hardship was more prevalent among those wishing for support, underlining the bidirectional relationship between financial stress and mental health problems (30). While financial strain may contribute to poor mental health, mental health problems can in turn undermine work productivity and economic performance (31), creating a vicious cycle. Thus, financial relief measures and mental health support may be mutually reinforcing and should be considered in integrated policy approaches.

The qualitative content analysis of support wishes provided valuable insights into the specific forms of support desired by farmers. The largest proportion of responses referred to non-psychotherapeutic professional support, such as coaching, mediation, farm help, and domestic assistance. These responses underscore the centrality of work-related and structural stressors in farmers’ lives. Many farmers emphasized the need for holistic, farm-specific counseling that considers both operational and interpersonal challenges. The frequent wish for external support in managing farm work or caring for dependents reflects the limited flexibility and high workload typically associated with agricultural occupations (32). Such structural demands leave little room for self-care or participation in mental health programs and may increase the risk of chronic stress and burnout. Female farmers more frequently articulated specific needs for “professional help except mental support” (37.2% vs. 24.9%), including domestic assistance (2.5% vs. 0%). Women may face a greater cumulative burden from the dual demands of agricultural labor and domestic responsibilities, which could explain their stronger emphasis on external support (33). In contrast, men may be less likely to express or articulate support needs, potentially due to traditional masculinity norms and stigma related to help-seeking in rural contexts (34, 35).

Professional mental health support was the second most frequently mentioned category. Participants frequently emphasized structural barriers to access, such as high costs, bureaucratic hurdles, and limited availability. These findings point to systemic gaps in the mental health care infrastructure, particularly in rural and agricultural regions. Farmers’ wishes for affordable and easily accessible mental health services mirror previous findings from rural populations (36) and emphasize the need for low-threshold, geographically and economically accessible mental health care.

Work-related burdens were another prominent theme, with many participants expressing concern about excessive regulation, agricultural policy uncertainty, and bureaucratic pressure. These concerns not only contribute to psychological stress but also reduce perceived autonomy and satisfaction with agricultural work. Moreover, a considerable number of farmers lamented the lack of societal appreciation for their work, which may contribute to feelings of isolation or diminished self-worth (37). Public discourse and policy measures should thus aim to better recognize the contributions of farmers to the society and alleviate structural pressures.

Recreational activities, including vacations or health retreats, were also commonly mentioned. Many farmers expressed the desire for time off and temporary relief from farm responsibilities, yet simultaneously noted that such breaks were not feasible due to lack of replacement workers or financial constraints. These responses highlight the importance of integrating recreational and restorative activities into occupational health frameworks for agricultural workers. Programs that provide temporary replacement workers or subsidized health stays may represent viable avenues for supporting farmers’ mental health. Female farmers more frequently articulated wish for recreational activities (19.1% vs. 10.9%), which could be due to a higher burden due to dual demands of agricultural labor and domestic responsibilities (33).

Financial support, either through fair pricing mechanisms or direct subsidies, was mentioned by several participants as a precondition for improved mental well-being. These findings again emphasize the entanglement of financial insecurity and psychological distress (38). Ensuring the economic sustainability of farming through appropriate policy mechanisms may thus serves not only agricultural but also public health objectives.

Lastly, communication and social support emerged as important themes in some responses. Social support is widely recognized as a central protective factor in mental health, functioning both as a buffer against psychological distress and as a facilitator of recovery (39, 40). Different theories have been proposed to explain the positive associations between social ties and mental health. These include, the stress-buffering model, that posits that perceived emotional and instrumental support can mitigate the negative effects of stressors (41). Another conceptualization is the main effect model, where an overall beneficial effect of social support is assumed to improve mental well-being (41). According to the Conservation of Resources Theory (42), social support is vital because it helps people retain and restore valuable resources, making it a central factor in coping with stress and maintaining mental health.

In the context of agriculture, where individuals often work in isolation and under chronic pressure, social connection may serve as one of the few readily available forms of support. However, structural and cultural barriers—such as long working hours, geographic distance, and mental health stigma—can erode informal support systems. Farmers who lack regular social interaction may be at heightened risk of developing or maintaining psychological distress. Our findings suggest that mental health interventions should not only address individual symptoms but also aim to build social capital and strengthen interpersonal networks within rural communities.

From a policy perspective, enhancing opportunities for peer exchange, community-based programs, and local support groups may represent cost-effective and culturally sensitive ways to promote mental well-being in agricultural populations. Initiatives that foster a sense of belonging and mutual understanding—such as farmer cafés, peer mentoring, or structured discussion groups—can help reduce feelings of loneliness, normalize help-seeking behavior, and increase mental health literacy. Moreover, integrating psychosocial support components into existing agricultural advisory services may improve uptake and reduce stigma. Given the well-established link between social support and mental health outcomes, bolstering farmers’ social connectedness is not only a theoretical imperative but also a practical and actionable goal for future health promotion strategies.

## 5 Limitations

Several limitations should be considered when interpreting the results of this study. First, the cross-sectional design precludes any conclusions about causal relationships between support wishes and mental health indicators. Second, the reliance on self-report data may have introduced biases, such as social desirability bias or underreporting of psychological distress and help-seeking behavior—especially given the stigma still associated with mental health problems in agricultural communities. Third, the open-ended question on support wishes yielded a wide range of responses with varying levels of detail, and the categorization process, although conducted rigorously, inevitably involved a degree of subjective interpretation. Another important limitation concerns sample representativeness: although recruitment was broad and involved multiple agricultural organizations, the self-selection of participants may have led to a participation bias, with possibly higher response rates among individuals already concerned about mental health. Furthermore, the findings reflect expressed support wishes, but it remains unclear to what extent these translate into actual behavior change, service uptake, or improved mental health outcomes.

## 6 Conclusion

In conclusion, this study highlights a substantial unmet need for mental health support among Austrian farmers and provides a nuanced understanding of the types of support desired. Structural, work-related, and psychosocial stressors converge to impact mental well-being, and farmers articulate diverse support needs that go beyond traditional mental health care. A multipronged approach—addressing financial strain, providing practical help, expanding accessible mental health services, and promoting societal appreciation—is essential to foster mental well-being in this vulnerable occupational group.

## Data Availability

The raw data supporting the conclusions of this article will be made available by the authors, without undue reservation.
